# Clinical response to antibiotics in indigenous versus non-indigenous children under 5 years old with community-acquired pneumonia in Otavalo, Ecuador

**DOI:** 10.1590/0037-8682-0038-2020

**Published:** 2020-06-22

**Authors:** Susana Eulalia Dueñas Matute, Eduardo Antônio Donadi, Altacílio Aparecido Nunes, Edson Zangiacomi Martinez

**Affiliations:** 1 Universidad Central del Ecuador, Quito, Ecuador.; 2 Universidade de São Paulo, Faculdade de Medicina de Ribeirão Preto, Ribeirão Preto, SP, Brasil.

**Keywords:** Antibiotics, Cultural characteristics, Ecuador, Indigenous people, Parturition, Pneumonia

## Abstract

**INTRODUCTION:**

Community-acquired pneumonia (CAP) is an important cause of morbidity and mortality worldwide. This study compares the clinical response to antimicrobials between indigenous and non-indigenous Kichwa children under 5 years old with CAP in Otavalo, Ecuador.

**METHODS:**

All children with CAP who met the inclusion criteria and were admitted at the San Luis de Otavalo Hospital between March 2017 and June 2018 were evaluated.

**RESULTS:**

No significant differences were observed in clinical responses between indigenous and non-indigenous children.

**CONCLUSIONS:**

The improved healthcare access of the Otavalo’s Kichwa population may have contributed to the observed clinical response to CAP treatment.

Community-acquired pneumonia (CAP) is an acute disease of the lung parenchyma that affects non-hospitalized patients and is characterized by the appearance of fever and/or respiratory symptoms together with pulmonary infiltrates on chest radiography^1^. According to the World Health Organization (WHO), CAP is responsible for 15% of all deaths of children aged below 5 years^2^. In 2014, 5.9 million children under the age of 5 years died of preventable causes; among them, 43% died of CAP, malaria, or diarrhea - all diseases that can be prevented by immunization. CAP caused by bacteria should be treated with antibiotics; however, only one-third of the children with pneumonia receive the antibiotics they need^2^ ^,^ ^3^. Latin-American countries show an incidence of 919 cases of CAP per 100,000 children under 5 years old; the rate in children under 2 years old is even higher (i.e. 1,412 deaths per 100,000), whereas that in children older than 6 years is lower (539 deaths per 100,000). According to data from the National Institute of Statistics and Census (Instituto Nacional de Estadística y Censo), CAP is the third highest cause of mortality among children aged 1-5 years in Ecuador^4^. In 2010, the infant morbidity rate due to CAP was 210 deaths per 100,000 inhabitants, with a mortality rate of 9.7% compared to general mortality rate^5^. The main risk factors are poverty, overcrowding, use of firewood inside the home, and an incomplete immunization schedule. The child’s clinical response is an indicator of the therapeutic efficacy of CAP treatment, which should be considered together with other indicators such as age, comorbidities, etiological agents, pneumonia severity, low weight, breastfeeding status, vaccination, and cultural habits. Due to their poor life conditions, indigenous people may be more susceptible to developing infectious disorders. On the other hand, cultural traditions including the use of herbal medicine prior to medical consultation have led Ecuadorian pediatricians to believe that indigenous children respond more rapidly to antibiotics.

The objective of the present cross-sectional study was to assess whether Kichwa indigenous children younger than 5 years old attending the San Luis de Otavalo Hospital for CAP treatment have a different clinical response to antimicrobials than non-indigenous children. Ecuador is a South American country with a population of 16,776,977 inhabitants in 2018. It has three continental regions (Sierra, Costa, Amazonia) and an insular region, all of which are divided into 24 provinces. The indigenous population corresponds to 7.23% of the Ecuadorian population, with approximately 1 million individuals grouped into 14 nationalities and 19 indigenous ethnicities. The history of the Kichwa peoples of Ecuador dates back to the pre-Incan times, when more than 60 different peoples lived together^6^. The Kichwas currently correspond to 90% of the indigenous population of the Sierra and Amazonia regions, which are affected by poverty, marginalization, and social repudiation^7^. Otavalo is located in the province of Imbabura, north of Ecuador. The city comprises an area of 579 km^2^ at an altitude of 2,565 m above sea level, being a distant 110 km from the capital Quito. In contrast to other indigenous cities, Otavalo has a very strong indigenous cultural tradition in which indigenous mayors pay attention to the community’s sanitary conditions. For instance, the community has 330 indigenous health professionals and the San Luis de Otavalo Hospital has implemented the use of intercultural care for indigenous mothers, thus allowing the incorporation of community health agents (midwives) for hospital care. As a result, the maternal mortality rate at San Luis Hospital Otavalo has been zero of each 100,000 live births since 2010^8^. This model offers alternatives according to the cultural conceptions of the indigenous people, including a delivery room with space for the preparation of medicinal infusions^9^.

All indigenous and non-indigenous children under 5 years old who received antimicrobials to treat CAP at San Luis de Otavalo Hospital between March 2017 and June 2018 were evaluated. The Clinical Practice Guide on Pneumonia Acquired in the Community of the Ministry of Public Health was applied to select the study group. Patients with fungal infections were excluded. After signing an informed consent form, the parents of the children answered a sociodemographic questionnaire. Clinical information was obtained from the children’s medical charts. The participants were divided into three groups according to age - infants (1 month to 1 year old), toddlers (1-2 years old), and preschoolers (2-5 years old) - a classification commonly used in Latin-American countries^10^. Children with inadequate and adequate birth weight were defined as those with body weight at birth <2,500 g versus those >2,500 g, respectively. Vaccination was considered complete if the children were fully immunized according to the vaccination scheme of the Ministry of Public Health of Ecuador. The families of antibiotics used to treat CAP were divided into first, second, and third generations. Fisher’s exact test was used to determine the main associations between ethnicity (i.e. indigenous or non-indigenous) and other study variables at a statistical significance level of 0.05. The statistical calculations were performed using R software, version 3.6.0 (The R Foundation for Statistical Computing, Vienna, Austria). The study was approved by the Research Ethics Committee of the Central University of Ecuador (Quito, Ecuador).

The demographic characteristics of the 106 children (72 indigenous [67.9%]) are shown in Table 1. When indigenous children were compared to non-indigenous children, there were no significant intergroup differences in sex, age group, vaccination scheme, or water supply. No non-indigenous children were born at home, whereas 11.1% of the indigenous children were. The indigenous children lived in houses with better sanitation than the non-indigenous children. No respondent reported using firewood for cooking. Table 2 compares the clinical variables of the indigenous and non-indigenous children. We found no statistically significant evidence of an association between ethnicity and survival at hospital discharge (either alive or dead), type of antibiotic used, and length of hospital stay. Comparison of the length of hospital stay between indigenous and non-indigenous children as a discrete variable instead of a dichotomized one within 6 days revealed no statistically significant evidence of an association (non-parametric Wilcoxon’s test, *P* = 0.06). However, non-pharmacological therapies were more often used for indigenous children, since their families have medicinal plant and other traditional indigenous beliefs. 

**TABLE 1:** Participant characteristics by ethnic group.

	Indigenous		Non-indigenous		Total		P value
	(n = 72)		(n = 34)		(N = 106)		
	n	%	n	%	n	%	(a)
							
**Sex**							0.20
Male	48	66.7	18	52.9	66	62.3	
Female	24	33.3	16	47.1	40	37.7	
**Age**							0.63
Infant	43	59.7	17	50.0	60	56.6	
Toddler	21	29.2	12	35.3	33	31.1	
Preschool	8	11.1	5	14.7	13	12.3	
**Birth weight**							0.50
Adequate	63	87.5	32	94.1	95	89.6	
Inadequate	9	12.5	2	5.9	13	10.4	
**Birthplace**							0.05
Hospital	64	88.9	34	100.0	98	92.5	
House	8	11.1	0	0.0	8	7.5	
**Residence**							
Rural	52	72.2	13	38.2	65	61.3	
Urban	20	27.8	21	61.8	41	38.7	
**Breastfeeding duration**							0.51
6 months	27	37.5	9	26.5	36	34.0	
12 months	27	37.5	16	47.1	43	40.6	
24 months	18	25.0	9	26.5	27	25.0	
**Vaccinations**							1.00
Complete	56	77.8	27	79.4	83	78.3	
Incomplete	16	22.2	7	20.6	23	21.7	
**Water supply**							1.00
No	4	5.6	2	5.9	6	5.7	
Yes	68	94.4	32	94.1	100	94.3	
**Sewerage system**							<0.01
No	30	41.7	24	70.6	54	50.9	
Yes	42	58.3	10	29.4	52	49.1	
							

(a) Fisher´s exact test.

**TABLE 2:** Clinical responses by ethnic group.

	Indigenous		Non-indigenous		Total		P value
	(n = 72)		(n = 34)		(N = 106)		
	n	%	n	%	n	%	(a)
							
**Status at discharge**							1.00
Alive	71	98.6	34	100.0	105	99.1	
Dead	1	1.4	0	0.0	1	0.9	
**Antibiotic type**							0.26
None	1	1.4	0	0.0	1	0.9	
First-generation (b)	24	33.3	12	35.3	37	34.9	
Second-generation (c)	12	16.7	11	32.4	22	20.8	
Third-generation (d)	11	15.3	4	11.8	16	15.1	
Double combination	13	18.1	6	17.6	24	22.6	
Triple combination	11	15.3	1	2.9	6	5.7	
**Length of hospital stay**							0.60
≤6 days	57	79.2	29	85.3	86	81.1	
>6 days	15	20.8	5	14.7	20	18.9	
**Nonpharmacological therapy**							0.03
Yes	29	40.3	12	35.3	41	38.7	
No	35	48.6	11	32.4	46	43.4	
Unknown	8	11.1	11	32.4	19	17.9	

(a) Fisher´s exact test; (b) ampicillin or gentamicin; (c) ampicillin plus sulbactam, or amoxicillin plus clavulanic acid; (d) ceftriaxone or cefotaxime.

In the present study, the mean length of hospital stay did not differ between the ethnic groups. However, this finding is not corroborated by a study showing that indigenous children of the Pacific Asia region with respiratory illnesses had longer stays than non-indigenous children^11^ and required longer oxygen therapy and antibiotic treatment durations. The risk factors known to be closely related to hospital admissions due to CAP among indigenous children include low weight, use of firewood for cooking, low maternal educational level, and poor socioeconomic conditions^12^. In the present study, these associations were not identified since the indigenous children were more likely to have an adequate birth weight and the use of firewood for cooking is rare due to the common use of gas. 

Despite the WHO guidelines on the appropriate use of antibiotics in children with CAP, this study found that these treatment guidelines are not considered at the time of their prescription. According to the WHO, first-generation antibiotics should be used only for younger infants with CAP who have been exclusively breastfed for the first 6 months, are up to date on their immunizations, and had an adequate birth weight. This was also observed in a study conducted in Dhaka, Bangladesh, in which parenteral ceftriaxone was used in approximately 50% of the children with CAP, followed by cefotaxime plus amikacin (17%)^13^. Therefore, local public health authorities and regulatory agencies must pay more attention to the irrational use of antibiotics to prevent the development of antimicrobial resistance.

Some limitations of the present study may be noted. San Luis de Otavalo Hospital does not have an electronic medical record system; thus, the collection of data from medical charts had to be done manually. Another important limitation involves the statistical analysis and relatively small sample size. With regard to the associations between the variables of interest (Table 1 and Table 2), many frequencies less than or equal to zero were observed, making it difficult to measure the associations (e.g. odds ratios). For that same reason, it was not possible to fit multiple logistic regression models to study the relationship between ethnicity and other variables of interest adjusted for potential confounders. Therefore, the statistical analysis was limited to determining *p* values ​​on association tests. The classification criterion of antibiotic type may be considered another limitation of the present study since a more adequate assessment could be performed using the classical criteria for categories of antimicrobial use by Kunin et al.^14^ or its modification proposed by Kawanami & Fortaleza^15^. However, these criteria are dependent on the patient’s clinical status, and the retrospective data collection used in the present study provided insufficient information to classify the patients according to antibiotic used. 

Despite these limitations, this study allowed us to show that the indigenous Kichwa population of Otavalo lives in social/cultural conditions similar to those of the non-indigenous population living in the same area, who sometimes even have better sanitation standards than the non-indigenous population. Although these results cannot be extrapolated to other Kichwa communities in Ecuador with different sanitary conditions, the present study showed that sociopolitical measures focused on interculturality can positively affect the health of both mother and child as observed in the Otavalo community. It is important to understand the Andean people’s worldview and their perceptions, concepts, and rituals regarding the health-disease process. This knowledge, together with public strategies to effectively improve primary care activities, can help reduce the morbidity and mortality rates of the indigenous child population.
